# Wrist, but Not Back, Isometric Contraction Induced Widespread Hypoalgesia in Healthy Participants

**DOI:** 10.3389/fpain.2021.701830

**Published:** 2021-08-04

**Authors:** Catherine Mailloux, Timothy H. Wideman, Hugo Massé-Alarie

**Affiliations:** ^1^Centre interdisciplinaire de recherche en réadaptation et intégration sociale, Université Laval, Quebec City, QC, Canada; ^2^Lethbridge-Layton-Mackay Rehabilitation Centre, School of Physical and Occupational Therapy, McGill University, Montreal, QC, Canada; ^3^Département de réadaptation, Centre interdisciplinaire de recherche en réadaptation et intégration sociale, Université Laval, Quebec City, QC, Canada

**Keywords:** exercise-induced hypoalgesia, pressure pain threshold, isometric contraction, back muscles, low back pain

## Abstract

**Objective:** Exercise may reduce pain sensitivity. This phenomenon called exercise-induced hypoalgesia is observed in different types of exercises and involves the activation of endogenous pain modulation systems. Although the effect of limb exercise on pain sensitivity has often been tested, few studies explored the impact of back exercises that are often used to treat low back pain. The main objective is to measure the effect of back-muscle exercise on pain sensitivity and compare it to the effect of a limb-muscle exercise.

**Methods:** Twenty-three participants who were pain-free performed a 4-min wrist flexion isometric contraction followed by a 4-min low back extension, separated by a 20-min break. Pressure pain thresholds were tested at two low back (S1 spinous process, lumbar erector spinae muscle) and two wrist (capitate bone, wrist flexor muscles) sites before and after each exercise. For each exercise, sites were considered as remote or local in relation to the muscles contracted during the exercise. An independent sample of 11 participants was recruited to confirm the influence of low back extension on pain sensitivity.

**Results:** Wrist exercise induced a larger increase in pain sensitivity than back exercise at the remote site. Only wrist exercise induced a hypoalgesia effect at both the local and the remote sites. Back exercise induced a similar effect in the independent sample.

**Conclusions:** This study showed that back and wrist exercises induced a distinct effect on pain sensitivity in participants who were pain-free. The wrist exercise induced a systemic reduction in pain sensitivity (locally and remotely), whereas the back exercise did not. This differential effect may be present because wrist exercise induced most fatigue compared with the back exercise.

## Introduction

Chronic low back pain (CLBP) is the leading cause of disability worldwide (1). Many clinical guidelines and reviews recommend exercise therapy as a first-line treatment and for routine use (2–5). Exercises targeting trunk muscles are frequently used in research and in clinical practice to normalize spine motor control, force, and/or endurance. There is low-to-moderate quality evidence that they are more effective than minimal intervention to improve pain and disability [for a review, see (6)]. Although specific changes in motor control (7, 8) and muscle size (9) can occur after exercises in LBP, systematic reviews have failed to associate these changes with changes in pain and disability (10–12). This suggests that other factors could explain the efficacy of exercises targeting trunk muscles.

Multiple studies have suggested that exercises could directly impact pain sensitivity (13). Indeed, aerobic, isometric, and resistance exercises induce widespread hypoalgesia (13–15). Studies based on animals and humans suggest that this exercise-induced hypoalgesia (EIH) involves the activation of multiple endogenous pain modulation systems, such as opioidergic and serotonergic systems, which are known to have antinociceptive effects at peripheral and central levels (15–19). A meta-analysis reported that isometric and dynamic resistance exercises induced the largest reduction in pain sensitivity in healthy adults compared with aerobic exercises (13). Different activities and physiological factors have been related to EIH [e.g., physical activity level (20), autonomic function (21–24), and fatigue (25, 26)], but evidence of relationship remains scarce. Although there is clear evidence of the effect of isometric contraction exercises on pain sensitivity in healthy subjects (13), most of the studies tested contractions of the upper or lower limb muscles.

Only a few studies investigated the effect of trunk muscle contractions on pain sensitivity in healthy subjects. Results are inconclusive for exercises that focus on the contraction of low back muscles, with one study demonstrating a slight increase in pain sensitivity over the low back region (27), whereas two others reported an absence of modulation in pain sensitivity (26, 28). Therefore, isometric contractions of different muscle groups may uniquely influence pain sensitivity. For example, recently, our group showed that stretching of the back muscles induced a widespread reduction in pain sensitivity, whereas stretching of wrist muscles reduced pain sensitivity only at local sites (29). Although different types of exercises were tested in this study, it is possible that trunk and limb exercises have distinct effects on pain sensitivity. Considering that exercises using back-muscle contraction are recommended to treat individuals with CLBP, there are important clinical implications to understand if and how they influence pain sensitivity. The first step is to test the influence of these exercises among healthy participants.

The objectives of this study were (i) to determine whether there is a difference in EIH at local and remote sites induced by a back-muscle exercise compared to that of a wrist-muscle exercise in healthy participants, (ii) to determine the effect of wrist and back exercises on pain sensitivity, and (iii) to explore the association between potential activity and physiological factors (e.g., perceived muscle fatigue) and EIH.

## Materials and Methods

### Participants

Thirty-four participants (18 females; 29.0 ± 10.3 years old) were recruited in two different studies. The sampling method was by convenience with emails sent to Laval University community and by solicitation at the research center. The determination of the selection criteria was based on consensus statements by the EUROPAIN and NEUROPAIN consortia for the quantitative sensory testing studies to ensure validity of the data (30). Exclusion criteria were (1) pain lasting 3 months or longer, located anywhere in the body, or any pain at the wrist or low back areas; (2) severe problem relating to health (such as cancer, major rheumatoid, cardiac, neurologic, or psychiatric disease); (3) LBP lasting more than 7 days in the last 6 months; (4) consultation with a health professional because of LBP in the last 6 months; (5) current bilateral wrist or forearm pain; and (6) current pregnancy and/or delivery in the last year. Participants who were currently taking medications like antidepressants, opioids, neuroleptics, anticonvulsive drugs, or steroids were also excluded. This study was approved by the local ethics committee (CIUSSS-*Capitale Nationale*, project #2019-1547), and all the participants provided informed written consent prior to the experimentation.

#### Study 1: Difference Between EIH After Wrist and Low Back Isometric Contractions

Twenty-four healthy participants (12 women; 28.3 ± 11.0 years old) aged between 19 and 62 years were recruited between December 2018 and June 2019 to participate in study 1. For objective 1, the sample size was based on the published results from our group on the effect of stretching exercises on pressure pain threshold (PPT) (29). Based on an effect size of *d* = 0.636, α = 0.05, and 1-β = 0.80 (two-tail matched *t*-test), 22 participants were required to observe a significant difference between exercises. For objective 2, based on an effect size of *d* = 0.85 [median effect size in a systematic review on the effect of exercise on pain sensitivity for studies using a similar methodology (13)], α = 0.05, and 1-β = 0.80 (two-tail matched *t*-test), 13 participants were required to observe pre-post difference in the PPT.

### Study Design

**Figure 1:**
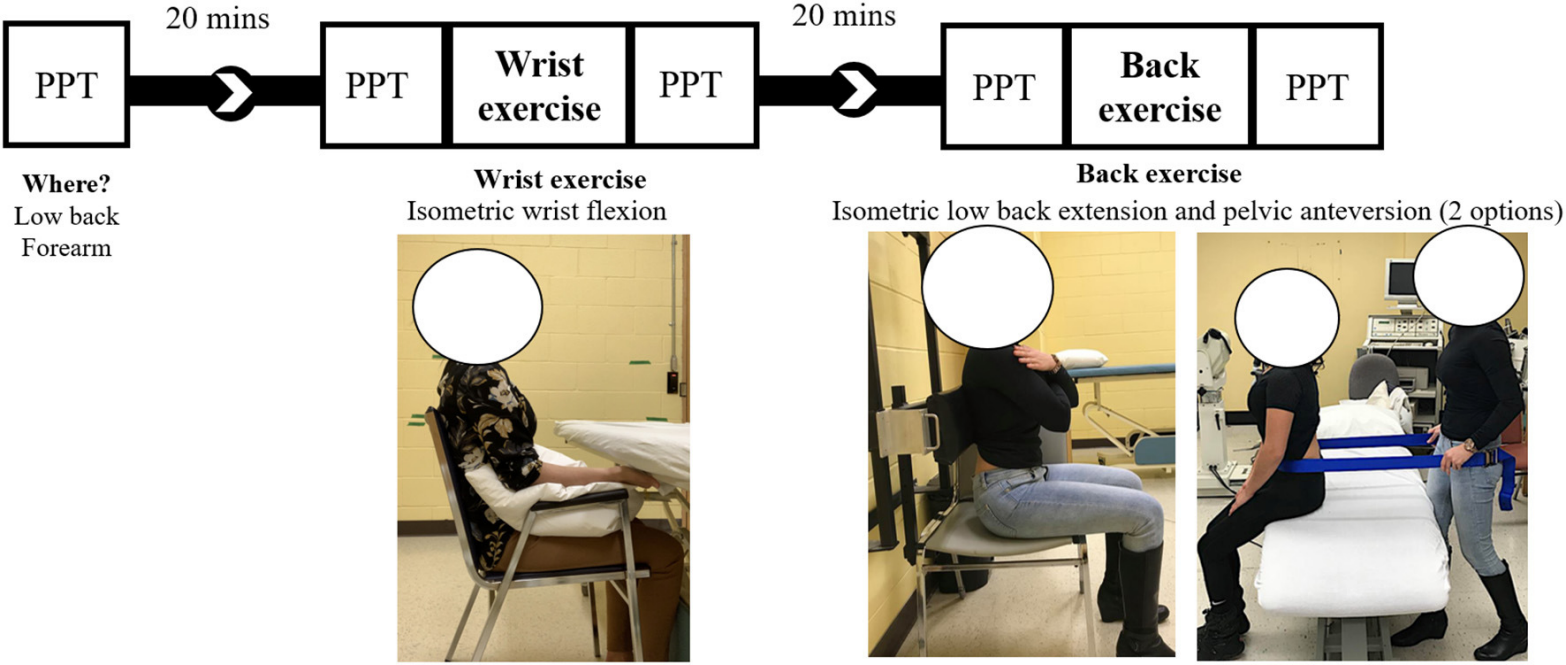
Representation of the study design and exercises. PPT: Pressure pain threshold.

Figure 1 illustrates the different steps of the study. The session started with a baseline PPT evaluation followed by the performance of two isometric exercises in a nonrandomized manner: (i) wrist flexion and (ii) low back extension. PPTs were measured before and after each bout of exercise (pre/post design). Breaks of 20 min were imposed between the baseline and the wrist exercise and between wrist and low back exercises. These 20-min recovery intervals were used as a washout period to avoid a carryover effect, as performed in similar studies (31–33). To reduce the likelihood that randomization of the exercise order introduced variability in the effect, wrist flexion was always tested first. To determine whether the nonrandomized exercise order impacted the results of the back exercise, a second sample of participants was recruited to confirm the validity of the back-exercise results (see study 2 below for details). Results from the baseline and prewrist flexion were used to measure the reliability of PPT. The minimal detectable change for group (MDC_gr_) ranges between 35.1 and 48.2 kPa for back sites (6.7–8.8%) and between 28.9 and 29.8 kPa for wrist sites (8%) and was already published (34).

### Electromyography and Maximal Voluntary Contraction

Electromyography was used as feedback to help maintain a minimal contraction level during the execution of the motor task. EMG wireless sensors (Trigno™ Wireless EMG System, Delsys, USA) were placed on (i) a lumbar erector spinae (LES), 2–3 cm laterally to L4/L5, and on (ii) wrist flexor (WF) muscles, 10 cm distally from the medial humeral epicondyle on a line from the medial epicondyle to the styloid process of radius over the muscles bulk of the WFs.

Maximal voluntary contractions of LES and WF muscles were tested three times with a 1-min break in between. For back-muscle exercise, we noticed in pilot experiments that some participants were unable to activate LES using back extension (e.g., flexion of the spine occurred during the resisted back extension). Thus, the activation of LES was first tested using two different methods: (i) resisted isometric anterior pelvic tilt and (ii) lumbar isometric extension (Figure 1). The method producing the highest EMG activity at LES was used for MVC and as the back-muscle exercise (see the description on *back-muscle exercise*) to be able to adequately activate the LES. This method was used to ensure that the exercise selected successfully activated LES.

### Isometric Exercises

Participants had to contract the muscle tested (displayed as a bar graph visual feedback on a computer monitor) at a 25% MVC [indicated as a red line on the bar graph (0.25-s RMS window length)] for 4 min or until exhaustion. A meta-analysis reported that isometric contractions of longer duration and of moderate intensity demonstrated the largest effects on pain sensitivity in healthy adults (13). Two 5-s breaks were allowed for all participants during the exercises in the presence of pain or muscle fatigue. Participants were asked to minimize the activation of the muscles that were not targeted by the specific exercise (e.g., avoiding contraction of back muscles during wrist flexion). Visual appreciation of the exercise by the assessor and online EMG recording ensured a preferential contraction of the muscle targeted by the exercise and relaxation of the non-tested muscle.

#### Wrist Exercise

Participants sat on a chair. The arms were positioned along the trunk with the elbow flexed at 90°. The forearm was supported by a pillow, and the hand was placed under the table. Participants pushed against the table with the forearm in a supine position (Figure 1).

#### Back Exercise

As described above, the back exercise was performed using either resisted (i) anterior pelvic tilt or (ii) back extension, as depicted in Figure 1. For anterior pelvic tilt, a strap was placed around the pelvis of the participant at the anterior superior iliac spine level. The assessor was positioned behind the participant and resisted the movement. Indications were given to participants to push against the strap, without lifting the feet off the ground (35). For back extension, participants pushed against an adjustable pad fixed on the wall. The pad was positioned at the mid-inferior thoracic level. Participants were asked to push against the pad in a posterior and inferior direction, without lifting the feet off or pushing on the ground to specifically activate the back rather than hip muscles. Sitting position was chosen over a prone-lying position to reduce the contribution of hip extensors and to be more specific to LES muscles (36).

### Pressure Pain Threshold

Pressure pain threshold was assessed as the static measure of pain perception (37), and all measures were evaluated in a standardized environment (same room with stable conditions regarding light, temperature, and noise). PPT testing order and side were randomized independently of dominance. If one side (wrist or low back) presented deficiencies other than pain, the other side was tested. PPT measures were first tested on the calf or the thigh for familiarization with the procedure. PPT measures were assessed with a handheld digital algometer (1-cm^2^ probe—FPIX, Wagner Instruments, Greenwich, CT, USA) for 22 participants and with a handheld dial algometer (1-cm^2^ probe—FPK, Wagner Instruments, Greenwich, CT, USA) for 2 participants. Since the FPK algometer does not allow us to measure between 0 and 1 kg/cm^2^, the FPIX algometer was used for the remaining participants. A rate of ~0.5 kg/cm^2^/s was applied, at two back and two upper limb sites (one muscle and one bony site): (i) LES and (ii) WF (same sites than EMG sensors) and (iii) S1 spinous process and (iv) dorsal aspect of the wrist on the capitate bone (WD). All sites were located and marked before testing. All assessments of the back and the forearms were done in a prone-lying position, with a pillow under the abdomen and in a sitting position with the arm supported, respectively. Standardized verbal instructions were given according to the recommendations from the German Research Network on Neuropathic Pain (DFNS) (38). Instructions were freely translated into French. PPT was measured three times with a 1-min break between measurements. To reduce the variability of the measurement and reduce the impact of potential outlier, a fourth measure was taken if the following two conditions were met: (1) the SD of the three measures was larger than 1 kg/cm^2^ and (2) one measure was outside the mean ± SD interval. The same criteria were applied to the four values to determine if three or four data were kept for analysis. Because of the accuracy limit of the FPIX algometer, PPT data above 11 kg/cm^2^ were considered as 11 kg/cm^2^. Four measures at S1 and three trials at LES exceeded this limit. For the FPK, PPT data between 0 and 1 kg/cm^2^ were considered as 1 kg/cm^2^ (four measures at WD−2 first participants). This may have led to a small underestimation of the effects of exercise on pain sensitivity. PPT measures were transformed from kg/cm^2^ to kPa (1 kg/cm^2^ = 98.07 kPa) to facilitate comparisons with the PPT literature. In healthy individuals, the intrasession reliability of the PPT at the low back and the wrist was demonstrated to be excellent (34, 39, 40).

### Activity and Physiological Factors Related to EIH

Additional activity and physiological factors that may influence the response to exercise were measured. All participants completed the self-administered French version of Global Physical Activity Questionnaire (GPAQ) to measure the physical activity level (41, 42). The French version of GPAQ provides acceptable reliability and validity (43). The perception of the intensity of the MVC (perceived MVC) was estimated by each participant in the first 30 s of each exercise. The Borg-modified scale (44) was also used to evaluate local (fatigue of the muscle contracted) and global (general fatigue) perceived exertion levels (Borg_local_ and Borg_general_) at the end of each exercise. Systolic and diastolic blood pressures (SBP and DBP), heart rate (HR) pre- and post-exercises, and their differences (Δ) were collected using a tensiometer (UA-767PAC, Lifesource, A&D Medical, Mississauga, ON), considering that past studies suggested an interaction between the autonomic/cardiovascular function and EIH (21–24).

### Data Preparation and Statistical Analysis

SPSS software was used for statistical analysis (IBM SPSS 25 for Mac, Armonk, NY, USA). Descriptive statistics (mean and SD) of the demographic data includes age, gender, handedness, body mass index (BMI), dominant site assessed, GPAQ score, and back extension exercise used.

#### Testing Assumptions for the Use of Parametric Analysis

Shapiro–Wilk's test and visual appreciation of histogram and normal Q–Q plots were used to assess the normality of PPT distributions. The presence of outliers was assessed by the inspection of a boxplot for values >1.5 times the interquartile range above the third quartile or below the first quartile (representing a 99.3% confidence interval). Considering that most PPT datasets presented non-normal distribution and numerous outliers and that data transformations (e.g., log, square root) did not allow us to normalize distributions, Wilcoxon signed-rank tests were used for within-participant analyses.

#### Data Preparation

Since no difference was present between the muscle and bony sites in the same area, data were pooled (WD-WF and S1-LES). Then, the percentage of change for pooled PPT (%PPT) at local and remote sites for each exercise was calculated [(*PPT*_*post*_−*PPT*_*pre*_)/*PPT*_*pre*_ ].

#### Statistical Analysis per Study Objectives

##### First Objective

A Wilcoxon signed-rank test was used to compare if low back and wrist isometric contractions induced a different impact, independently, on local and remote sites for both %PPT.

##### Second Objective

To test the individual effect of back and wrist exercises on pain sensitivity, a Wilcoxon signed-rank test compared the difference between pre- and post-exercise PPTs. The mean and median percentage of PPT and the effect size for the Wilcoxon-signed rank test (r = Z / $\sqrt{N}$, where N is the number of observations) were calculated to inform on the effect and to facilitate comparisons with the literature.

##### Third Objective

A Wilcoxon signed-rank test compared if wrist and back exercises impacted differently on activity and physiological factors associated with EIH (Borg_local_, Borg_general_, SBP, DBP, HR, GPAQ, and perceived MVC). Spearman's rank-order correlation tested the relationship between %PPT and the factors associated with EIH.

#### Assessment of Carry-Over Effect

To test for the presence of a carryover effect, a Wilcoxon signed-rank test was used to evaluate whether there was a difference in the absolute PPT between prewrist exercise and preback exercise for back and wrist sites.

### Study 2: Validation of the EIH Following Back Exercise

An additional project was realized to determine whether the non-randomized study design (same exercise order for all participants) may have impacted EIH following back-muscle contraction. This was particularly important since a carryover effect (significant difference between prewrist flexion and pre-low back extension) was present at back sites after the wrist muscle contraction (see Section **Results**).

#### Participants

An additional sample of 11 participants who were healthy and pain-free were recruited [six women, mean age ± (SD): 30.4 (8.1)].

#### Study Design

All participants attended one session during which they performed the back-muscle exercise only, with PPT evaluation before and after. One subject was excluded from the analysis for back-site PPT because the pressure recorded exceeded the superior limit of the algometer for all measurements.

#### Statistical Analysis

The demographics of the participants of studies 1 and 2 were compared using the Mann–Whitney *U* test.

The objective of study 2 was to confirm whether local and remote EIH following the back-muscle exercise were similar to study 1 using an independent sample of participants. The Mann–Whitney *U* test tested whether the %PPT differed between samples [tested second (study 1) or tested first (study 2)]. A *P*-value below 0.05 was considered statistically significant. Data are presented as medians [min, max] unless otherwise stated.

## Results

### Study 1

#### Participants

A total of 24 participants were eligible for the study. For one participant, it was impossible to record EMG of LES above the background noise due to the difficulty in voluntarily activating LES and due to the presence of the adipose tissue over LES. Thus, data from 23 participants were analyzed. Descriptive characteristics are summarized in Table 1. The local perceived exertion level was significantly higher for the wrist exercise compared with that for the back exercise [wrist: mean (SD) = 4.78 (2.19), back: 2.91 (1.82), *p* = 0.001]. The global perceived exertion level and changes in systolic and diastolic blood pressures, as well as in heart rate, and the perceived level of MVC were comparable for the two exercises. All participants completed the 4-min exercises without break, except one participant who reported exhaustion at 177s for the wrist exercise. A Wilcoxon-signed rank test revealed a significant difference between the PPT at the prewrist exercise [521.7 (253.0; 945.4) kPa] compared to that at the preback exercise [570.7 (273.6; 938.5) kPa; *z* = −2.581, *p* = 0.010], suggesting a carryover effect at this site. No significant difference was noted at the forearm [345.2 (166.7; 580.6) kPa vs. 376.6 (168.7; 614.9) kPa; *z* = −1.780, *p* = 0.075].

**Table 1:** Descriptive characteristics of participants ($\bar{X}\pm$ SD).

	**Study 1 (*****n*** **=** **23)**		**Study 2 (*****n*** **=** **11)**		***P^**B**^***
Age, years	28 ± 11		30 ± 8		0.153
Gender—Male, %	52		45		0.897^c^
Handedness, Right, %	83		91		1.000^d^
BMI	22.50 ± 2.38^†^		23.26 ± 1.87		0.289
Dominant site assessed, %	39		55		0.475^d^
GPAQ (METS)	2,763 ± 2,018		3,207 ± 3,009		0.856
Low back exercise options—anterior pelvic tilt (%)	70		82		0.682^d^
	**Wrist exercise**	**Back exercise**	***P** ^**a**^*	**Back exercise**	***P** ^**b**^*
Borg_local_	4.78 ± 2.19	2.91 ± 1.82	*0.001*	4.27 ± 1.95	0.077
Borg_general_	1.35 ± 2.26	1.17 ± 1.77	0.972	3.27 ± 2.66	0.060
**ΔBP**
Systolic	+5.87 ± 28.34	+3.39 ± 9.63	0.626	−0.55 ± 10.08	0.363
Diastolic	+5.83 + 6.69	+4.26 ± 17.05	0.260	0.18 + 5.90	*0.013*
ΔHR	−6.17 + 7.60	−3.30 ± 16.99	0.465	−6.00 + 5.88	0.971
Perceived MVC	40.53 ± 16.43^*^	31.76 ± 18.26^*^	0.163	37.05 ± 10.05	0.264

*BMI, body mass index; GPAQ, global physical activity questionnaire; METS, metabolic equivalent; Δ, difference post–pre-exercise; BP, blood pressure; HR, heart rate; Perceived MVC, percentage of maximal voluntary contraction perceived*.

* *n = 18*.

† *n = 21*.

a *A Wilcoxon signed-rank test compared the difference between wrist and back exercises*.

b *The Mann–Whitney U test compared the difference between back exercise effects from studies 1 and 2*.

c *Chi-squared test*.

d *Fisher's exact test*.

#### PPT

**Figure 2:**
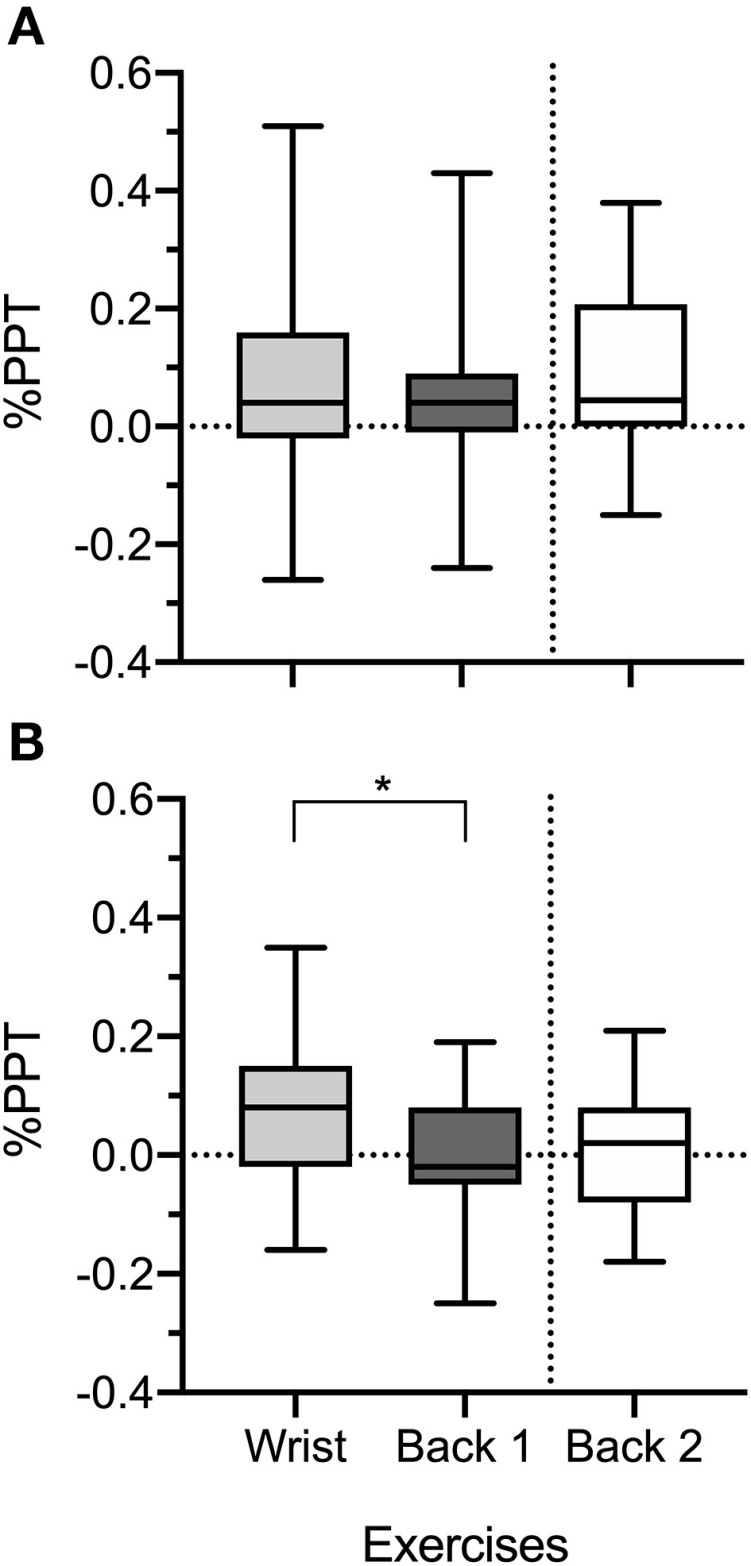
Percentage of change of pressure pain thresholds (%PPT) at local **(A)** and remote **(B)** sites following wrist exercise and back exercises for studies 1 and 2. *p < 0.05.

A Wilcoxon-signed rank test revealed a significantly larger %PPT at remote sites following wrist exercise [8 (−16, 35)%] when compared with %PPT after back exercise [−2 (−25, 19)% | *z* = −2.27, *p* = 0.02; Figure 2A]. No significant difference was detected at local sites [wrist-exercise: 4 (−26, 51)%; back-exercise: 4 (−24, 43) % | *z* = −1.08, *p* = 0.28; Figure 2B]. Significant differences between pre- and post-PPT were only observed for the wrist exercise (*p* = 0.02 at the local site, *p* = 0.03 at the remote site), suggesting a significant widespread EIH following this exercise (Table 2). No significant effect was detected for the back exercise for both studies 1 and 2. Note that a positive %PPT represents a decrease in pain sensitivity.

**Table 2:** Exercise effects on pressure pain thresholds at local and remote sites.

	**Exercises**	**Sites**	**PPT (kPa)** ** median (min; max)**	**PPT (kPa)** ** mean (SD)**	**%PPT** ** median** ** (min; max)**	**%PPT** ** mean (SD)**	***r***	***P***
Study 1 (*n* = 23)	Back	Local			4 [−24; 43]	4 (13)	0.215	0.144
		Pre	570.8 [273.6; 938.5]	589.7 (197.2)				
		Post	625.7 [277.5; 1012.1]	605.4 (200.5)				
		Remote			−2 [−25; 19]	1 (10)	0.026	0.855
		Pre	376.6 [168.7; 614.9]	366.7 (108.7)				
		Post	362.8 [181.4; 585.5]	367.9 [115.5]				
	Wrist	Local			4 [−26; 51]	8 (16)	0.334	*0.023*
		Pre	345.2 [166.7; 580.6]	354.4 (119.2)				
		Post	377.6 [158.9; 612.9]	379.1 (124.7)				
		Remote			8 [−16; 35]	7 (12)	0.318	*0.031*
		Pre	521.7 [253.0; 945.4]	536.7 (183.1)				
		Post	573.7 [279.5; 876.7]	568.4 (181.0)				
Study 2 *(n=11)*	Back	Local			4 [−15; 38]	8 (12)	0.353	0.114
		Pre	345.7 [96.1; 556.2]	328.1 (130.1)				
		Post	343.8 [97.3; 769.3]	360.9 (179.6)				
		Remote			2 [−18; 21]	0 (9)	0.133	0.534
		Pre	287.5 [119.4; 634.5]	310.4 [140.6]				
		Post	280.0 [97.9; 684.5]	319.4 [165.3]				

*SD, standard deviation*.

*r effect size for the Wilcoxon-signed rank test*.

*p Wilcoxon signed-rank test*.

#### Activity and Physiological Factors Associated With EIH

Correlations between %PPT and factors associated with EIH are reported in Table 3. Significant associations were found between perceived MVC and %PPT at the remote site following wrist exercise (ρ = 0.642; *p* = 0.003). Also, GPAQ was significantly correlated with %PPT at the local site for back exercise (ρ = 0.454, *p* = 0.03), and ΔDBP was negatively associated with %PPT at the remote site for back exercise (ρ = −0.424, *p* = 0.03). Overall, 3 out of 28 correlations were significant.

**Table 3:** Correlations between %PPT and exercise variables.

**Outcomes**	**Exercises**	**Sites**	**Borg_**LOCAL**_**	**Borg_**GEN**_**	**ΔSBP**	**ΔDBP**	**ΔHR**	**GPAQ**	**%MVC**
%PPT	Wrist exercise	Local	−0.230	−0.180	−0.162	−0.209	−0.207	0.032	0.286^a^
		Remote	−0.044	−0.062	−0.081	−0.258	−0.101	0.242	0.642^**^ ^a^
	Back exercise	Local	−0.262	−0.250	−0.339	−0.187	0.109	0.454^*^	−0.231^b^
		Remote	−0.297	−0.049	−0.243	–0.452^*^	−0.163	0.216	0.027^b^

*Δ difference post-/pre-exercise; SBP, systolic blood pressure; DBP, diastolic blood pressure; HR, heart rate; GPAQ, Global physical activity questionnaire; %MVC percentage of maximal voluntary contraction perceived*.

* *p < 0.05*,

** *p < 0.01*.

a *n = 19*.

b *n = 17*.

### Study 2

#### Comparison of EIH Following Back-Muscle Exercise

Sample characteristics were comparable between studies (Table 1). The %PPT for back exercise realized in studies 1 and 2 was not significantly different at local [study 1: 4% (−24, 43%); study 2: 4% (−15, 38%) | *U* = 102, *z* = −0.511, *p* = 0.630; Figure 2A] and at remote sites [study 1: −2% (−25, 19%); study 2: 2% (−18; 21%) |*U* = 123.5, *z* = −0,111, *p* = 0.913 |; Table 2 and Figure 2B]. Mean and median %PPT and the effect size are reported in Table 2.

## Discussion

This study compared the effect of back and wrist exercises on pain sensitivity and explored the association between potential activity and physiological factors thought to be involved in EIH and the modulation of pain sensitivity following exercises. Our results demonstrated that the wrist flexion produced a significant increase in pain sensitivity at local and remote sites. In addition, this reduction in pain sensitivity at remote sites was larger after wrist exercise than after back exercise.

### Differential Effect of Wrist and Back Exercises on PPT

Our results indicate a greater reduction in pain sensitivity at remote sites after wrist exercise when compared with %PPT after back exercise. The widespread effect following wrist exercise suggests that the magnitude of EIH may differ depending on the muscle group tested. This difference occurred despite the execution of both exercises at the same intensity of muscle contraction. However, the local perceived exertion level (Borg_local_) following wrist exercise was higher compared with that after the back exercise. This means that participants rated the wrist exercise as more fatiguing to perform at the level of the muscle contracted. One possible explanation is that the difference regarding fiber-type composition of the two contracting muscle groups may have influenced the modulation of pain sensitivity. For example, back muscles present a larger proportion of slow-twitch fibers (54–74%—type I: endurance muscle function) compared to fast-twitch fibers (21–53%—type II: phasic muscle function) (45–47). Conversely, wrist muscles are composed of 35–85% fast-twitch fibers compared to 15–65% slow-twitch fibers (48). Thus, the duration and intensity of contraction may not have fatigued enough low-threshold motor units of the back muscles to engage high threshold motor units that are thought to be involved in the induction of hypoalgesia (49–51). Studies testing the effect of exercises involving back-muscle contraction seem to support this hypothesis. Two studies investigating the influence of the same repetitive lifting task presented conflicting results: an absence of pain sensitivity modulation across the low back area (28) and a reduction in pain sensitivity (27). The only difference in the study design was the duration of the task (i.e., 3 and 7 min). Finally, a third study that tested the effect of an isometric back exercise (120-s Biering–Soerensen test) reported no EIH at the back (26). This suggests that a longer duration of the back exercise could be necessary to induce muscle fatigue of back muscles and local hypoalgesia.

Reduction in pain sensitivity at remote sites suggests a systemic effect potentially driven by the activation of central endogenous pain modulation systems (31, 52, 53). As suggested by some authors, reduction in pain sensitivity at the remote sites following exercise involving limb-muscle contraction may be more related to a dose–response effect (14, 26). Similarly, we observed a widespread reduction in pain sensitivity following wrist exercise only, the most fatiguing task, but not following back exercise, which was less exhausting. This is in line with the literature where all but one of the studies testing back exercises did not induce a widespread reduction in pain sensitivity (26–28). In the study that observed hypoalgesia at the remote site, a widespread reduction in pain sensitivity was only present in women but not in men (26).

Although the literature and some of our results point toward muscle fatigue as a potential mechanism involved in EIH, some did not. For example, no significant association was present between the modulation of pain sensitivity and the perceived fatigue using the Borg-modified scale. It is possible that methodological considerations explain these differences. It is likely that the back exercises (anterior pelvic tilt or back extension) also recruited and fatigued the thoracic *erector spinae* (54). For example, Kuithan et al. (27) tested PPT modulation following repeated flexion tasks at 16 locations covering the lumbar area (from T12 to L5) and observed a larger reduction in pain sensitivity at cranial sites. Similarly, it is possible that our back exercises also induced a reduction in pain sensitivity at the lower thoracic and the higher lumbar spine due to the contraction of thoracic *erector spinae* during the back exercises. Also, it is unlikely that participants were able to distinguish whether the fatigue perceived at the back was coming from the lumbar or lower thoracic area. Thus, future studies exploring the effect of back exercise on pain sensitivity should use objective measures of fatigue (e.g., change in the slope of median frequency during contraction) and PPT at the level of the thoracolumbar junction in addition to that at the lumbar spine.

Using the data collected at baseline and before the wrist flexion task (see Figure 1), we measured that the percentage of the MDC_gr_ was ~8% at wrist sites and ~7–9% at back sites (34). This implies that the effect following wrist exercise (study 1) was close to or slightly over the natural variability of our technique. This suggests that wrist exercises induced a true increase in pain sensitivity. In study 2, the mean %PPT at the back following back exercise was around 8%, suggesting that a larger sample could have confirmed a local hypoalgesia effect induced by the back exercise. The smaller effect in study 1 could be explained by the carryover effect (i.e., PPT at the back sites was larger before the back contraction compared to that before the wrist contraction).

### Correlations With the Modulation of Pain Sensitivity Following Exercises

Modulation of pain sensitivity was not consistently associated with any of the factors tested. For example, we did not observe the association between %PPT and autonomic function (SBP, DBP, and HR). In the literature, blood pressure variation following exercise seems to be more correlated to the change in pain intensity and less correlated to pain sensitivity measures as PPT in clinical population (23, 24). In the present study, blood pressure post-exercise was assessed about 1–2 min after the completion of exercise due to logistic and environmental limitations. Considering that isometric exercises engage the cardiovascular system minimally, the time interval before retesting may have been enough to reverse blood pressure change.

### Perspective for Experimental and Clinical Studies

The fact that the immediate (i) wrist exercise induced systemic hypoalgesia (including the low back area) and (ii) low back exercise did not induce hypoalgesia could highlight the relevance of prescribing peripheral/remote and/or global exercises to maximize the alleviation of pain in CLBP and not only target the painful area if the objective is to influence pain sensitivity. However, our results remain to be validated in CLBP. Indeed, smaller EIH compared to healthy controls was observed in participants with CLBP (27, 28). This difference could be explained by the fact that EIH relies, in part, on the activation of endogenous pain modulation systems [e.g., opioidergic and serotoninergic systems—for a review, see Rice et al. (15)] and that some patients with CLBP present with alteration in neural areas involved in pain modulation (55, 56). Future research is needed to better understand brain areas involved in EIH and potential alterations in CLBP.

### Limitations

Results need to be interpreted while considering some methodological limitations. The exercise order was not randomized. However, considering that back contraction influenced pain sensitivity in a similar manner in studies 1 and 2, it suggests that the absence of randomization did not explain the differences between the types of exercises. The sample size of study 2 was small and may have inflated the risk of type II errors. Although it seems possible for the modulation of pain sensitivity at the local site following back exercises (study 2 mean %PPT = 8%), it appears unlikely for the remote site (study 2 mean %PPT = 0%). Thus, it seems that back exercise did not induce a widespread hypalgesia, at least under the parameters used in this study. It is possible that the effect of back exercise was slightly reduced because of the carryover effect present at the back sites. Although a 20-min washout period was used as performed in similar published studies (31–33), it is suggested that the effect may last up to 30 min (13). Future studies should consider using a washout period of at least 30 min. The use of multiple statistical tests could have inflated the likelihood to have type I errors. Taking into account that young adults were mostly recruited as participants for this study, our results cannot be generalized to older adults. A different algometer was used for two participants. However, this potential bias was considered negligible considering the within-participant design of the study and because results of the statistical analysis remain similar after removing these two participants. Finally, although exclusion criteria were exhaustive, other factors such as sleep restriction, ovulation and luteal phases of the menstrual cycle in women, and caffeine intake could have affected pain sensitivity.

### Conclusions

This study showed that back and wrist exercises induced a distinct effect on pain sensitivity in participants who were pain-free. The wrist exercise induced a systemic reduction in pain sensitivity, whereas the back exercise did not. This differential effect may be present because wrist exercise induced more fatigue compared to the back exercise. Future studies are required to test this hypothesis. The systemic response following wrist exercise could be used in the CLBP population to determine the presence of an alteration in central pain processing, whereas the back exercise could be used to explore the impact of contracting a painful area on pain processing.

## Data Availability Statement

The raw data supporting the conclusions of this article will be made available by the authors, without undue reservation.

## Ethics Statement

The studies involving human participants were reviewed and approved by CIUSSS-Capitale Nationale, project #2019-1547. The patients/participants provided their written informed consent to participate in this study.

## Author Contributions

HM-A, TW, and CM conceived the idea for the paper. CM handled the recruitment of participants and the collection of data. CM and HM-A performed the data analysis. All authors were involved in drafting the article and revising it critically for important intellectual content.

## Conflict of Interest

The authors declare that the research was conducted in the absence of any commercial or financial relationships that could be construed as a potential conflict of interest.

## Publisher's Note

All claims expressed in this article are solely those of the authors and do not necessarily represent those of their affiliated organizations, or those of the publisher, the editors and the reviewers. Any product that may be evaluated in this article, or claim that may be made by its manufacturer, is not guaranteed or endorsed by the publisher.
